# Serum Cytokines in Young Pediatric Patients with Congenital Cardiac Shunts and Altered Pulmonary Hemodynamics

**DOI:** 10.1155/2016/7672048

**Published:** 2016-08-30

**Authors:** Leína Zorzanelli, Nair Yukie Maeda, Mariana Meira Clavé, Vera Demarchi Aiello, Marlene Rabinovitch, Antonio Augusto Lopes

**Affiliations:** ^1^Heart Institute, University of São Paulo School of Medicine, São Paulo, SP, Brazil; ^2^Pró-Sangue Foundation, São Paulo, SP, Brazil; ^3^Stanford University School of Medicine, Stanford, CA, USA

## Abstract

*Background and Objective. *Inflammation is central in the pathogenesis of pulmonary hypertension. We investigated how serum cytokines correlate with clinical features, hemodynamics, and lung histology in young patients with pulmonary hypertension associated with congenital cardiac shunts.* Design. *Prospective, observational study.* Methods and Results*. Patients (*n* = 44) were aged 2.6 to 37.6 months. Group I patients (*n* = 31) were characterized by pulmonary congestion and higher pulmonary blood flow compared to group II (*p* = 0.022), with no need for preoperative cardiac catheterization. Group II patients (*n* = 13) had no congestive features. At catheterization, they had elevated pulmonary vascular resistance (5.7 [4.4–7.4] Wood units·m^2^, geometric mean with 95% CI). Cytokines were measured by chemiluminescence. Macrophage migration inhibitory factor (MIF) was found to be inversely related to pulmonary blood flow (*r* = −0.33, *p* = 0.026) and was higher in group II (high pulmonary vascular resistance) compared to group I (high pulmonary blood flow) (*p* = 0.017). In contrast, RANTES chemokine (regulated on activation, normal T cell expressed and secreted) was characteristically elevated in Group I (*p* = 0.022). Interleukin 16 was also negatively related to pulmonary blood flow (*r*_*S*_ = −0.33, *p* = 0.029) and was higher in patients with obstructive vasculopathy at intraoperative lung biopsy (*p* = 0.021).* Conclusion*. Cytokines seem to be important and differentially regulated in subpopulations of young patients with cardiac shunts.

## 1. Introduction

Inflammation and altered immune processes are involved in all forms of pulmonary vascular disease underlying pulmonary hypertension, particularly, pulmonary arterial hypertension (PAH) [1]. Perivascular inflammatory elements have been reported in several etiologies of PAH, from the idiopathic form to PAH associated with autoimmune disorders [2–4]. Inflammatory mediators have been investigated in pediatric PAH, including PAH associated with congenital heart disease (PAH-CHD) [5–9]. Studies generally include patients over a wide age range, from infancy to adolescence, with multiple etiologies of PAH.

There has been scarce literature on the role of inflammatory mediators in the specific population of young pediatric patients with CHD and pulmonary hypertension who are being considered for surgical repair of their cardiac anomalies. These patients generally have communications between cardiac chambers or the great arteries leading to increased pulmonary blood flow and pressure. Progressive remodeling of pulmonary arteries causes elevation of pulmonary vascular resistance (PAH-CHD). Structural and functional pulmonary vascular abnormalities are sometimes early, unsuspected, and progressive even in young individuals treated for their cardiac lesions in a timely manner [10].

There are reasons for studying inflammation and immune response in young patients with congenital cardiac communications undergoing surgery. Inflammatory cells, mediators, and related molecules have been shown to be closely associated with phenomena that occur early in pulmonary microvasculature, such as medial hypertrophy, muscularization of distal arteries, and intimal proliferative lesions [5, 11, 12]. These abnormalities constitute the anatomical basis for increased pulmonary vasoreactivity that occurs postoperatively as a result of vasospastic stimuli. Although the incidence of the so-called postoperative pulmonary hypertensive crises has decreased to less than 1% due to contemporary practice in privileged communities, the mortality has been estimated at 20–30% [13]. Pulmonary hypertension has been identified as a major contributor to prolonged mechanical ventilation and length of stay in hospital [14]. After discharge, some patients remain at a higher risk of persistent pulmonary hypertension despite successful surgery.

We therefore planned this study to investigate inflammatory mediators in young pediatric patients being considered for surgical repair of congenital cardiac communications. We wished to investigate how serum cytokines and related molecules correlate with patterns of clinical presentation, pulmonary hemodynamics, and pulmonary vascular abnormalities assessed by intraoperative lung biopsy.

## 2. Methods

### 2.1. Patients and Clinical Presentation

The study population consisted of young pediatric subjects (up to the age of 3 years) with congenital cardiac shunts admitted to the Heart Institute, University of São Paulo, Brazil, for surgical repair of their anomalies. From March 2012 to March 2015, 44 patients with nonrestrictive cardiac communications were enrolled, as they were suspected to have at least moderate elevation of pulmonary artery pressure. These patients correspond to 20–25% of all pediatric subjects with cardiac shunts undergoing surgery in the institution. After complete noninvasive evaluation, 31 patients were considered as suitable for surgery with no need for cardiac catheterization (study group I, clinical features of pulmonary overcirculation with pulmonary congestion). The remaining 13 patients presented with features that suggested higher levels of pulmonary vascular resistance: relatively late presentation with no history of failure to thrive, small-sized heart with no significant enlargement of left cardiac chambers, and absence of pulmonary congestion despite the size of the cardiac communication and bidirectional shunting. Preoperative cardiac catheterization was considered as necessary (study group II). Patients with these features generally correspond to less than 10% of pediatric subjects with cardiac shunts treated in the institution. Subjects with smaller septal defects not associated with pulmonary hypertension and those with advanced pulmonary vasculopathy precluding surgery were excluded. Neonates, patients under intensive care, and those with relevant comorbidities or extracardiac syndromes other than Down syndrome were also excluded. Written informed consent was obtained from the parents before inclusion. The study protocol was approved by the institutional Scientific and Ethics Committee, CAPPesq #0502-11.

### 2.2. Pulmonary Hemodynamics and Vascular Abnormalities

We used the following parameters to characterize hemodynamic and structural changes in pulmonary circulation: (1) pulmonary blood flow estimated noninvasively by transthoracic echocardiography, (2) pulmonary vascular resistance assessed by cardiac catheterization, and (3) morphology of pulmonary vessels assessed by direct analysis of lung biopsy specimens.

#### 2.2.1. Doppler Echocardiography

Transthoracic echocardiography was performed in all patients to define cardiovascular anatomy and assess the severity of pulmonary hypertension. The direction of flow across the cardiac communications and the size of the left cardiac chambers were taken into account when classifying patients as group I or group II. Left-to-right shunting (exclusive or predominant) in the presence of an enlarged left heart was considered as indicative of increased pulmonary blood flow (group I) whereas bidirectional shunting with a relatively small-sized left heart was suggestive of heightened pulmonary vascular resistance (group II). Of the parameters that were recorded, the characteristics of pulmonary venous flow were taken as an indirect estimate of total pulmonary blood flow. At least two pulmonary veins were selected and examined in all patients (same observer, LZ). The velocity-time integral of pulmonary venous blood flow was obtained (VTI_PV_, cm), and a mean value per patient was calculated. VTI_PV_ has been shown to be inversely related to pulmonary vascular resistance [15]. The pulmonary-to-systemic blood flow ratio (Qp/Qs) was also estimated [16], but it was considered less accurate in some cases due to the presence of a communication distal to the aortic and pulmonary valves (patent ductus arteriosus).

#### 2.2.2. Cardiac Catheterization

Invasive assessment of pulmonary hemodynamics was considered only for patients suspected to have higher levels of pulmonary artery pressure and vascular resistance on the basis of the clinical history, physical examination, and echocardiographic evaluation. Catheterization was carried out under general anesthesia and mechanical ventilation. Pulmonary and systemic blood flow were determined separately using the Fick method. However, to avoid error due to the use of assumed oxygen consumption while calculating blood flow [17], we decided to express the magnitude of pulmonary hemodynamic abnormalities by calculating the pulmonary-to-systemic vascular resistance ratio (PVR/SVR), with no need for considering the level of oxygen consumption.

#### 2.2.3. Lung Biopsy Assessment

Lung biopsies are not performed routinely in our institution. In this study, lung biopsy specimens were collected intraoperatively in selected cases: from group II patients if the surgeon considered the procedure low risk on an individual basis and from group I patients if the elevation of pulmonary artery pressure represented a problem during weaning from cardiopulmonary bypass. Thus, lung tissue was available for analysis in 13 of our patients (9 and 4 individuals from group II and group I, resp.). Specimens were collected with the airways distended, fixed in buffered formalin, and subjected to routine histological processing. Four-micrometer-thick sections were obtained and stained with hematoxylin-eosin and Miller's elastic stain. Vascular abnormalities were graded according to the Heath-Edward's classification for pulmonary vascular lesions associated with congenital heart disease [18]. Grades I and II correspond to isolated medial hypertrophy of the pulmonary arteries and intimal nonocclusive proliferation, respectively. Grades III through VI correspond to more advanced vascular abnormalities, including occlusive intimal proliferation, plexiform and dilated lesions, and necrotizing arteritis.

### 2.3. Inflammatory Mediators

**Figure 1:**
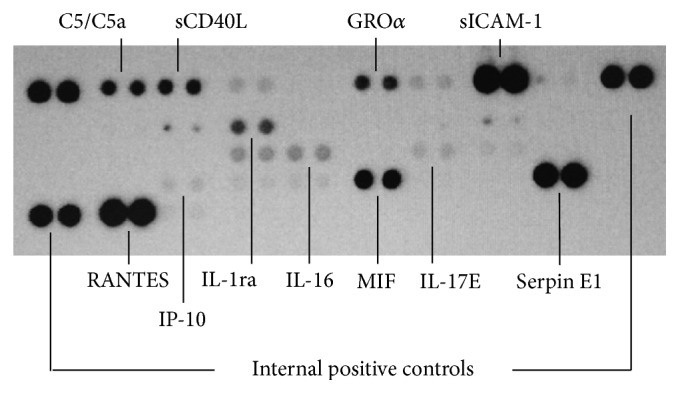
Representative immunoblotting for semiquantitative analysis of proteins investigated in the study. C5/C5a: complement component 5/5a; sCD40L: soluble CD40 ligand; GRO*α*: growth-regulated oncogene alpha; IL-16: interleukin 16; IL-1ra: interleukin-1 receptor antagonist; IL-17E: interleukin 17E; IP-10: interferon gamma-induced protein-10; MIF: macrophage migration inhibitory factor; RANTES: regulated on activation, normal T cell expressed and secreted; sICAM-1: soluble intercellular adhesion molecule-1.

Peripheral venous blood was collected, and serum samples were obtained and stored at −80°C. Cytokines and related proteins were analyzed using a human cytokine array (R&D Systems, Minneapolis, MN, USA). After protein immobilization on nitrocellulose membranes, processing (immunoblotting) was carried out using specific antibodies as previously described [19]. Chemiluminescence was used for semiquantitative detection. The average signal (pixel density) of the pair of duplicate spots representing each protein was normalized using internal controls. Of the 36 inflammatory mediators that were potentially measurable, we analyzed only the proteins that we detected with adequate signal in all patients (Figure 1). These included complement component 5/5a (C5/C5a), soluble CD40 ligand (sCD40L), growth-regulated oncogene alpha (GRO*α*), interleukins 16 and 17E (IL-16, IL-17E), interleukin-1 receptor antagonist (IL-1RA), interferon gamma-induced protein-10 (IP-10), macrophage migration inhibitory factor (MIF), regulated on activation, normal T cell expressed and secreted (RANTES), Serpin E1, and soluble intercellular adhesion molecule-1 (sICAM-1).

### 2.4. Statistical Analysis

Results involving numeric variables are expressed as geometric mean with 95% CI. For categorical variables, the number of patients is given, with percentages when appropriate. Differences between groups were analyzed using Student's* t*-test or the Mann–Whitney test for numeric variables or the Chi-square family of tests (including Fisher's exact test and the likelihood ratio) for categorical variables. Correlations were tested by calculating Pearson's coefficient (*r*) or Spearman's coefficient (*r*_*S*_). For regression analyses, logarithmic transformation (Ln) of the dependent variable was sometimes necessary to obtain adequate closeness to the normal distribution. In all tests a *p* value lower than 0.05 was considered statistically significant.

## 3. Results

Forty-four patients were enrolled, with an age range of 2.6 to 37.0 months. Cardiac anomalies were relatively simple, consisting of pretricuspid and posttricuspid defects in biventricular hearts. Demographic and diagnostic data of patients classified as group I or group II are shown in Table 1. Group II patients tended to be a little older and had lower systemic oxygen saturation, probably due to some degree of right-to-left shunting across the septal defects. Consistent with this, echocardiographic parameters (Qp/Qs ratio and VTI_PV_) pointed toward lower pulmonary blood flow in group II patients compared to group I (Table 1). At catheterization, they had a mean pulmonary arterial pressure of 52 (95% CI 45–59) mmHg, a pulmonary vascular resistance of 5.7 (95% CI 4.4–7.4) Wood units × m^2^, and a pulmonary-to-systemic vascular resistance ratio of 0.39 (95% CI 0.31–0.50).

### 3.1. Cytokines and Clinical Presentation

As shown in Table 2, MIF chemokine concentration was higher in group II, where patients were assumed to have higher levels of pulmonary vascular resistance, whereas RANTES chemokine was more closely related to group I, corresponding to subjects with clinical features suggestive of increased pulmonary blood flow. The level of GRO*α* chemokine was higher early in life and decreased with increasing age (Table 2). The tendency toward higher GRO*α* levels in group I was probably because group I patients were a little younger compared to group II. In contrast, IL-17E increased with increasing age (Table 2).

### 3.2. Correlations with Hemodynamic Parameters Assessed Noninvasively and Invasively

**Figure 2:**
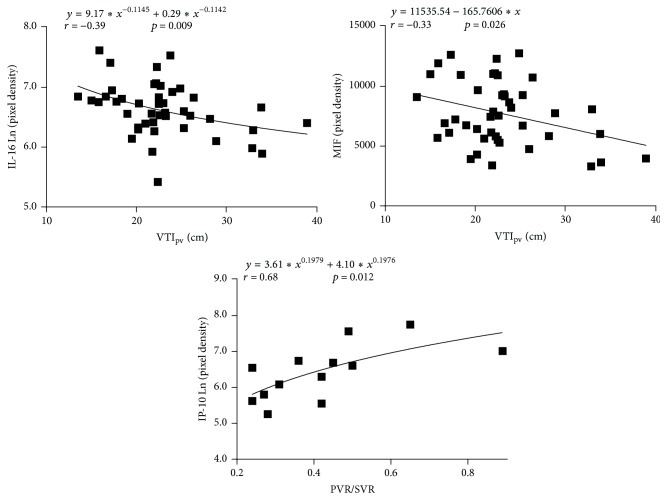
Serum concentration of inflammatory mediators (pixel density of chemiluminescence signal) at different levels of pulmonary blood flow (VTI_PV_, velocity-time integral of blood flow in pulmonary veins, echocardiographic assessment, *n* = 44) and pulmonary vascular resistance (PVR/SVR, pulmonary-to-systemic vascular resistance ratio, cardiac catheterization, *n* = 13). IL-16: interleukin 16; MIF: macrophage migration inhibitory factor; IP-10: interferon gamma-induced protein-1.

Consistent with the observation of higher MIF levels in group II patients was the negative correlation between MIF concentration and pulmonary blood flow estimated noninvasively by measuring VTI_PV_. A similar negative correlation was obtained for IL-16 (Table 2 and Figure 2). In group II, IP-10 chemokine level correlated positively with pulmonary vascular resistance (Table 2 and Figure 2). The correlation was negative for Serpin E1.

### 3.3. Cytokines and Lung Biopsy Findings

**Figure 3:**
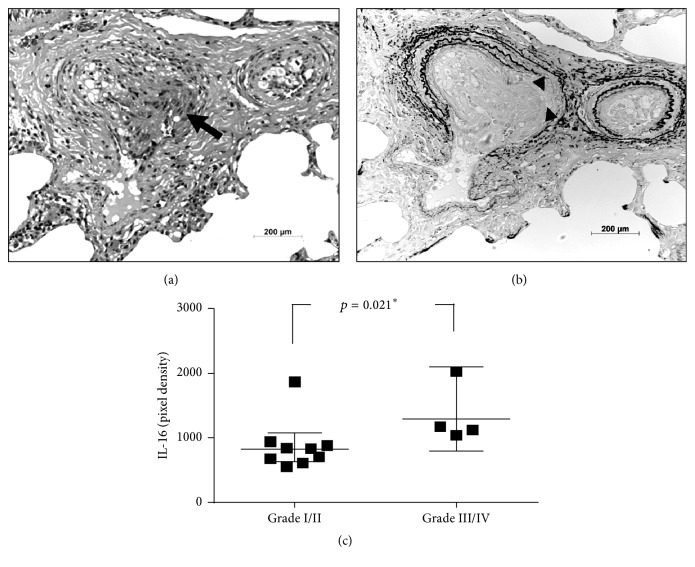
Lung biopsy findings and serum interleukin 16 (IL-16). (a) Advanced pulmonary vasculopathy (grade IV) in a young group II patient (11 months old). Preacinar arteries are shown, with focal intraluminal proliferation of endothelial cells (arrow) characterizing the early stage of a plexiform lesion. (b) Histological section of the same arteries, stained for elastic fibers. At the site where the endothelial proliferation takes place, there is absence of the internal elastic lamina, indicating local elastolysis (between the arrow heads). (a) and (b), respectively, hematoxylin-eosin and Miller's elastic stain; objective magnification 20x. (c) Serum levels of IL-16 (geometric mean with 95% CI) in patients at different degrees of pulmonary vascular abnormalities. ^*∗*^Mann–Whitney test.

Nine of the 13 patients who were subjected to intraoperative lung biopsy had pulmonary vascular lesions classified as grade I (5 cases) or grade II (4 cases). The remaining ones had more advanced lesions classified as grade III (3 patients) or grade IV (1 patient, Figure 3). Consistent with the negative correlation of IL-16 with pulmonary blood flow (VTI_PV_, Table 2 and Figure 2) was the observation of higher IL-16 levels in the subgroup of patients with more advanced pulmonary vascular abnormalities (Figure 3). No other significant correlations were observed.

### 3.4. Postoperative Pulmonary Vasoconstriction

Hemodynamic instability requiring specific therapeutic interventions occurred in 12 patients (6 individuals in each group), and there was a fatal outcome in two of them (group II). When considering only patients who had a lung biopsy specimen available for analysis (*n* = 13), clinically relevant postoperative pulmonary vasoconstriction occurred in 4 of 9 subjects with vascular lesions graded as I or II, and only one of 4 subjects with lesions graded as III or IV. Patients who died had grade I and grade II pulmonary vasculopathy. Preoperative oxygen saturation was lower in patients who had postoperative pulmonary vasoconstrictive episodes compared to those who did not (93% [91–95%] and 95% [94–97%], resp., *p* = 0.013). No other associations with preoperative variables were observed. The 27% incidence of postoperative pulmonary vascular tone instability was considerably higher than the 8% incidence in similar patients from our institution without clinical evidence of pulmonary hypertension.

## 4. Discussion

There is general agreement that patients with nonrestrictive cardiac communications and absence of clinical and echocardiographic evidence of pulmonary overcirculation may have pulmonary vascular changes that eventually preclude surgery [20]. In theory, any patients with group II characteristics, with a low VTI_PV_ and a high PVR/SVR ratio, would belong to this “high-risk” population. Of note, however, was the observation that postoperative pulmonary vasospastic episodes may occur even in subjects with group I characteristics, or those who have vascular abnormalities considered to be potentially reversible (Grade I). There seems to be a potential for occurrence of vascular tone instability leading to life-threatening hemodynamic disturbances all throughout the spectrum of pulmonary vascular changes. We did not test for direct correlations between preoperative cytokine levels and postoperative pulmonary vasoconstriction. Actually, pulmonary vascular tone instability is probably more closely related to perioperative than preoperative proinflammatory events. We focused essentially on the analysis of the preoperative scenario where pulmonary vascular remodeling is usually already present to a variable degree.

We identified associations between serum inflammatory mediators and patterns of clinical presentation, hemodynamics, and the morphology of pulmonary arteries, with potential pathophysiological significance. MIF and RANTES chemokines were easily analyzed, as they appeared in all of the membranes with a strong chemiluminescence signal (Figure 1). Higher concentration of MIF was a characteristic of group II patients, and MIF concentration correlated negatively with pulmonary blood flow estimated by transthoracic echocardiography (VTI_PV_). In contrast, RANTES was higher in group I, where patients had features compatible with pulmonary overcirculation with congestion.

MIF and RANTES chemokines are expressed by various cells and tissues under different conditions. MIF is a noncanonical ligand of CXCR2 and CXCR4 chemokine receptors and the CXCR2/CD74 complex. Its pleiotropic nature is illustrated by the activation of several signaling pathways that results in inhibition of endothelial cell apoptosis and induction of smooth muscle cell proliferation [1]. Its binding to the CXCR2/CD74 complex elicits chemotaxis and the arrest of mononuclear cells, which is central to inflammatory disorders and atherogenesis [21–23]. In clinical and experimental pulmonary hypertension, MIF is associated with a proinflammatory endothelial cell behavior, exaggerated recruitment of peripheral blood mononuclear cells, and increased smooth muscle cell growth [11, 24, 25]. RANTES is a CC chemokine shown to be involved in a number of biological events including chemotaxis, perivascular inflammation, and vascular response to injury [26, 27]. Of note, RANTES expression has been demonstrated in viral diseases [28], and it is widely appreciated that infants with pulmonary congestion due to cardiac shunts (group I patients in this study) are predisposed to respiratory infections. Thus, a possibility exists that repeated exposures to viral agents contribute to the development of a proinflammatory microenvironment, with expression of molecules known to influence vascular remodeling.

Another cytokine with potential pathophysiological implication is IL-16. Levels were higher in subjects with histological grades III and IV and correlated negatively with noninvasively estimated pulmonary blood flow. IL-16 is a lymphocyte chemoattractant factor well known for its role in immune responses, but there has been scarce literature on its role in vascular disorders [29]. IL-16 and its receptor CD4 are expressed in vascular smooth muscle cells. They induce migration and invasion of vascular smooth muscle cells in a p21WAF1-dependent manner, via the p38MAPK/Sp-1/MMP-9 pathway. For this reason, they are being considered as potential targets in the treatment of diseases such as atherosclerosis and restenosis [30]. The pathophysiological role of IL-16 and downstream signaling molecules in PAH have not been investigated yet.

We also observed significant correlations of IL-17E (positive) and GRO*α* (negative) with patient age, considered as an index of disease severity and progression in PAH-CHD [31, 32]. IL-17E and MIF are capable of eliciting a Th2-type immune response [33, 34] shown to be involved in experimental pulmonary vascular remodeling [35]. GRO*α* is a CXC chemokine known to play a central role in thrombin-induced angiogenesis associated with tumor growth [36]. A potential implication for the age-dependent decrease in GRO*α* is that reduction of distal pulmonary vessels does occur in pulmonary hypertension associated with CHD and probably precedes obliterative pulmonary vasculopathy [37].

In patients subjected to catheterization, there was a positive correlation between serum IP-10 and pulmonary vascular resistance. IP-10 is a CXC chemokine known to be involved not only in the endothelial-leukocyte interaction but also in the endothelial renin-angiotensin pathway [38]. It is involved in interferon-associated PAH [39] and plays a role in idiopathic PAH [40] and chronic thromboembolic pulmonary hypertension [41]. We also observed a negative correlation of Serpin E1 (formally, plasminogen activator inhibitor-1, PAI-1) with pulmonary vascular resistance. Despite its function as an inhibitor of fibrinolysis, Serpin E1/PAI-1 has a paradoxical protumorigenic role in cancer and has been shown to promote angiogenesis [42, 43]. Furthermore, Serpin E1/PAI-1 seems to be downregulated in pulmonary artery smooth muscle cells in idiopathic PAH [44].

In conclusion, correlations were observed between circulating levels of inflammatory mediators and patterns of clinical, hemodynamic, and histological presentation, suggesting that inflammation has a role in pulmonary vascular remodeling in young pediatric patients with cardiac shunts. However, in view of the relatively small number of patients in the study and so many correlations to be tested, we would like to focus on the observations that, in our opinion, are the most consistent ones. The other findings should be considered as exploratory at the present moment. Essentially, MIF chemokine was elevated in patients for whom cardiac catheterization was considered as necessary, and elevated pulmonary vascular resistance was confirmed. In contrast, RANTES chemokine was elevated in patients with congestive heart failure who were assigned to surgery with no need for catheterization. Furthermore, there seemed to be a positive correlation between IL-16 level and severity of pulmonary vascular lesions. Contemporary practice includes therapeutic measures that allow for the management of postoperative pulmonary vascular tone instability, although mortality rates are still high. Hopefully, in the future, specific targeted therapies will allow for stabilization or reduction of preoperative pulmonary vascular remodeling in this population.

## Figures and Tables

**Table 1:** Demographic and diagnostic data in patient groups.

	Group I (*n* = 31)	Group II (*n* = 13)	*p* value
Age (months)	8.6 (6.8–10.8)	12.7 (9.0–18.0)	0.068^*∗*^

Gender (M : F)	13 : 18	4 : 9	0.723^†^

Down syndrome (present : absent)	21 : 10	10 : 3	0.722^‡^

Weight (Kg)	6.01 (5.32–6.80)	6.73 (5.44–8.34)	0.537^*∗*^

O_2_ sat. (%)	96 (95–97)	92 (90–95)	0.011^*∗*^

Diagnosis^*‖*^			
A	1 (3%)	0 (0%)	0.573^§^
B	17 (55%)	6 (46%)	
C	13 (42%)	7 (54%)	

Echocardiography			
PAP (mmHg)	41 (34–49)	45 (38–53)	0.487^*∗*^
Qp/Qs	2.7 (2.3–3.0)	1.9 (1.4–2.6)	0.022^*∗*^
VTI_PV_ (cm)	23.3 (21.5–25.3)	20.2 (17.7–22.9)	0.080^*∗*^

Results are shown as geometric mean (95% CI) or number of cases and percentage.

^*∗*^Mann–Whitney test.

^†^Chi-square test.

^‡^Fisher's exact test.

^§^Likelihood ratio.

^*‖*^A: pretricuspid defects, B: posttricuspid defects except for atrioventricular septal defects, C: atrioventricular septal defects.

O_2_ sat.: peripheral oxygen saturation; Qp/Qs: pulmonary-to-systemic blood flow ratio; PAP: mean pulmonary arterial pressure estimated based on pulmonary insufficiency jet velocity; VTI_PV_: velocity-time integral of blood flow in pulmonary veins.

**Table 2:** Correlations of inflammatory mediators with clinical, echocardiographic, and hemodynamic parameters.

	Group I (*n* = 31)	Group II (*n* = 13)	*p* value	Age (*n* = 44)	VTI_PV_ (*n* = 44)	PVR/SVR (*n* = 13)
C5/C5a	2058 (1751–2419)	2504 (1953–3209)	0.169^*∗*^	*r*_S_ = −0.002 *p* = 0.988	*r*_S_ = −0.16 *p* = 0.301	*r*_S_ = −0.17 *p* = 0.583

sCD40L	2541 (2008–3216)	2046 (1403–2983)	0.252^*∗*^	*r*_S_ = −0.19 *p* = 0.227	*r*_S_ = 0.04 *p* = 0.809	*r*_S_ = −0.32 *p* = 0.287

GRO*α*	2005 (1592–2525)	1568 (1148–2141)	0.087^*∗*^	**r**_**S**_ = −0.46 **p** = 0.002	*r*_S_ = 0.05 *p* = 0.743	*r*_S_ = −0.06 *p* = 0.837

IL-16	748 (650–861)	782 (556–1100)	0.807^*∗*^	*r*_S_ = 0.13 *p* = 0.420	**r**_**S**_ = −0.33 **p** = 0.029	*r*_S_ = 0.12 *p* = 0.693

IL-1ra	1361 (1119–1655)	1366 (1014–1841)	0.949^*∗*^	*r*_S_ = −0.02 *p* = 0.923	*r*_S_ = 0.14 *p* = 0.372	*r*_S_ = 0.45 *p* = 0.121

IL-17E	150 (118–190)	158 (109–230)	0.728^*∗*^	**r**_**S**_ = 0.33 **p** = 0.028	*r*_S_ = −0.52 *p* = 0.735	*r*_S_ = 0.26 *p* = 0.398

IP-10	754 (575–988)	618 (390–977)	0.512^*∗*^	*r*_S_ = −0.07 *p* = 0.635	*r*_S_ = 0.20 *p* = 0.192	**r**_**S**_ = 0.70 **p** = 0.008

MIF	6706 (5889–7636)	8737 (7009–10891)	**0.017** ^†^	*r* = −0.09 *p* = 0.557	**r** = −0.33 **p** = 0.026	*r* = 0.03 *p* = 0.916

RANTES	71073 (63545–79492)	50228 (36485–69148)	**0.022** ^†^	*r* = −0.02 *p* = 0.882	*r* = 0.21 *p* = 0.166	*r* = −0.21 *p* = 0.482

Serpin E1	46149 (42119–50563)	43007 (35453–52172)	0.555^†^	*r* = −0.05 *p* = 0.764	*r* = −0.01 *p* = 0.959	**r = −0.64** **p = 0.019**

sICAM-1	47666 (41657–54541)	42635 (35434–51298)	0.238^†^	*r* = −0.09 *p* = 0.556	*r* = 0.13 *p* = 0.411	*r* = −0.21 *p* = 0.497

Protein levels are expressed as pixel density (geometric mean with 95% CI).

Reference values from 13 healthy pediatric subjects aged 10–38 months (data our laboratory): C5/C5a: 1726 (1434–3554); sCD40L: 2715 (2074–3554); GRO*α*: 1893 (1452–2467); IL-16: 630 (450–882); IL-1ra: 1513 (1089–2103); IL-17E: 228 (166–315); IP-10: 621 (412–936); MIF: 5547 (4487–6857); RANTES: 59692 (51567–69096); Serpin E1: 39810 (34442–46014); sICAM-1: 44185 (37272–52379).

“*r*” and “*r*_s_,” respectively, Pearson's coefficient of correlation and Spearman's coefficient of correlation.

^*∗*^Mann–Whitney test.

^†^Student's *t*-test.

PVR/SVR: pulmonary-to-systemic vascular resistance ratio; VTI_PV_: velocity-time integral of blood flow in pulmonary veins.

C5/C5a: Complement component 5/5a; sCD40L: soluble CD40 ligand; GRO*α*: growth-regulated oncogene alpha; IL-16: interleukin 16; IL-1ra: interleukin-1 receptor antagonist; IL-17E: interleukin 17E; IP-10: interferon gamma-induced protein-10; MIF: macrophage migration inhibitory factor; RANTES: regulated on activation, normal T cell expressed and secreted; sICAM-1: soluble intercellular adhesion molecule-1.
